# A New Role for Myosin II in Vesicle Fission

**DOI:** 10.1371/journal.pone.0100757

**Published:** 2014-06-24

**Authors:** Juan A. Flores, Santiago Balseiro-Gomez, Jose M. Cabeza, Jorge Acosta, Pilar Ramirez-Ponce, Eva Ales

**Affiliations:** Dpto. Fisiología Médica y Biofísica, Facultad de Medicina, Universidad de Sevilla, Seville, Spain; Institut Curie, France

## Abstract

An endocytic vesicle is formed from a flat plasma membrane patch by a sequential process of invagination, bud formation and fission. The scission step requires the formation of a tubular membrane neck (the fission pore) that connects the endocytic vesicle with the plasma membrane. Progress in vesicle fission can be measured by the formation and closure of the fission pore. Live-cell imaging and sensitive biophysical measurements have provided various glimpses into the structure and behaviour of the fission pore. In the present study, the role of non-muscle myosin II (NM-2) in vesicle fission was tested by analyzing the kinetics of the fission pore with perforated-patch clamp capacitance measurements to detect single vesicle endocytosis with millisecond time resolution in peritoneal mast cells. Blebbistatin, a specific inhibitor of NM-2, dramatically increased the duration of the fission pore and also prevented closure during large endocytic events. Using the fluorescent markers FM1-43 and pHrodo Green dextran, we found that NM-2 inhibition greatly arrested vesicle fission in a late phase of the scission event when the pore reached a final diameter of ∼ 5 nm. Our results indicate that loss of the ATPase activity of myosin II drastically reduces the efficiency of membrane scission by making vesicle closure incomplete and suggest that NM-2 might be especially relevant in vesicle fission during compound endocytosis.

## Introduction

Mast cells are specialized cells that respond to inflammatory signals by secreting large amounts of a wide range of inflammatory products. Some products, such as histamine, proteases and proteoglycans, are stored in cytoplasmic secretory vesicles and can be released by exocytosis upon stimulation, ensuring an immediate and maximal biological effect [1]. Early ultrastructural and electrophysiological studies showed that mast cell degranulation involves compound exocytosis, which, in addition to the fusion of vesicles at the plasma membrane, implicates the fusion of vesicles in either a multivesicular or sequential manner to allow the formation of degranulation channels [2], [3]. After secretory vesicles fuse with the plasma membrane, membrane retrieval must occur to maintain a constant cell size and facilitate the reuse of vesicular membrane components. Exocytosis in mast cells is followed by several forms of compensatory endocytosis, including kiss-and-run endocytosis and compound endocytosis, a mechanism by which the compound cavity, formed by the cumulative fusion of many secretory vesicles, is retrieved in a single membrane fission event [4]. In these modes of exo-endocytosis, the fused vesicles are not obliged to flatten, allowing the vesicles to be retrieved largely intact. These mechanisms do not require the effort of the invaginating membrane to form a deeply invaginated bud but do require the constriction and fission of the endocytic tubular neck to separate the vesicle from the plasma membrane and the inward movement of the vesicle into the cytosol [5], [6].

The mechanism by which vesicles separate from the plasma membrane is called membrane scission, and this process requires the large guanosine triphosphate hydrolase (GTPase) dynamin, curvature sensing/inducing N-terminal helix-containing Bin/Amphiphysin/Rvs (N-BAR) domain proteins and regulation by the actin cytoskeleton [7]. Accumulating evidence indicates a major role for actin in scission. Disruption of actin polymerization causes an increase in the number of endocytic vesicles that are unable to fully separate from the plasma membrane [8], [9], suggesting that actin polymerization is important for vesicle fission during endocytosis by providing direct mechanical forces. The formation of actin plumes at the constricted neck of the budding vesicle might provide the necessary force for pushing the bud deeper into the cytoplasm and may increase the strain on the stalk until it severs. However, a recent study demonstrated that actin polymerization does not provide direct mechanical forces for vesicle fission by analyzing the kinetics of the endocytic tubular membrane neck with capacitance measurements [10]. Other recent findings have indicated the involvement of actin-binding motor proteins in endocytosis, as in exocytosis. F-actin/myosin II interactions govern vesicle transport and are essential to ensure “normal” vesicular fusion kinetics, ending in full collapse [11]–[14]. F-actin/myosin II may influence fusion by exerting mechanical tension over the entire vesicle, or alternatively, this tension may only affect a hypothetical cytoskeletal scaffold required for pore dilation. By contrast, F-actin and NM-2 (or other myosin classes) molecules may directly affect the proteins that are responsible for the formation and expansion of the fusion pore [15]. Because NM-2 has emerged as a regulator of the exocytic fusion pore, NM-2 could be playing an unsuspected role in fission pore closure. Indeed, the depletion of NM-2 inhibits the scission of tubular carrier precursors that are positive for Rab6 [16], and the loss of NM-2 function causes defects in synaptic vesicle retrieval in response to stimulation [17], [18].

Rapid endocytosis can be distinguished kinetically from slower clathrin-mediated endocytosis using capacitance measurements [19] and by imaging techniques that assess the ability of a vesicle to release or uptake fluorescent luminal probes [20]–[22]. In the present study, we investigated the role of NM-2 in endocytosis by perforated-patch capacitance measurements and the activity-dependent markers FM1-43 and pHrodo Green dextran. By inducing the loss of NM-2 through pharmacologic inhibition, we found a new role for NM-2 in vesicle fission that predominantly affects the compound endocytic mode.

## Results

### NM-2 inhibition affects individual fusion and fission events

**Figure 1:**
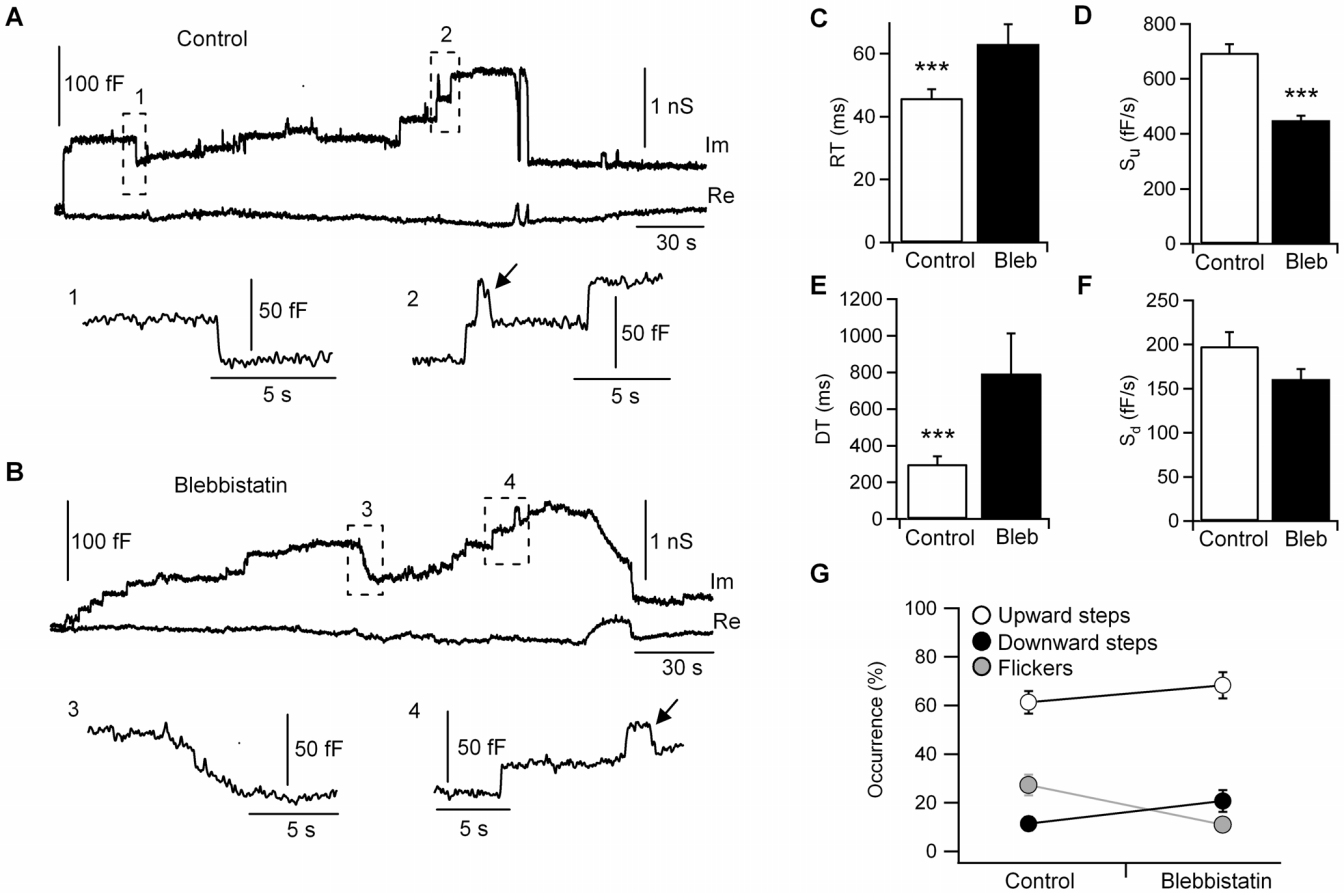
Inhibition of NM-2 activity through acute blebbistatin treatment slows the kinetics of exocytosis and endocytosis. Representative traces of capacitance steps from a control (A) and blebbistatin-treated cell (B). Re and Im represent the real and imaginary parts, respectively, of the admittance change. The steps labelled 1–4 at higher magnification show single downward steps, upwards steps and capacitance flickers (arrows). The kinetic parameters of the capacitance steps demonstrate that blebbistatin treatment affects the rise time (C) and upward-slope (D) to prolong the duration of the exocytic event. Blebbistatin also produces a increase in the decay time (E) without significantly affecting the downward-slope (F). The proportion of capacitance flickers (reversible fusion) decreased in blebbistatin-treated cells versus control cells (G) (p<0.05). Error bars, S.E.M. ***p<0.01.

The kinetics of fusion and fission of single vesicles were determined by perforated-patch capacitance measurements. Figures 1A and B show typical recordings from mast cells under control conditions and NM-2 inhibition, respectively. The cells were incubated in an extracellular solution containing blebbistatin (5 µM) for ∼ 10 min before the pipette was sealed onto the plasma membrane. Blebbistatin is a NM-2 ATPase inhibitor that has been found to bind to non-muscle myosin heavy chain IIA. Blebbistatin blocks the myosin heads by forming a complex with low affinity for actin [23], [24]. The upward and downward capacitance steps, which are associated with exocytosis and endocytosis, respectively, displayed slower kinetics in the cells treated with blebbistatin. To characterize the average fusion kinetics, we analyzed the rise time (RT) and upward-slope (S_u_) for each upward step. Blebbistatin-treated cells showed an increased rise time (RT = 63.1±6.2 ms; n = 582; 19 cells) (Figure 1C) with a reduced upward-slope (S_u_ = 450±16 fF/s) (Figure 1D) compared to control cells (RT = 45.9±2.8 ms; S_u_ = 696±31 fF/s; n = 497; 23 cells; p<0.001), suggesting slower individual release events. Similarly, the kinetics of fission were analyzed by calculating the decay time (DT) and downward-slope (S_d_). The decay time of the downward steps under NM-2 inhibition was also increased (DT = 793±220 ms; n = 237) (Figure 1E) compared to the control cells (DT = 300±43 ms; n = 293; p<0.001). However, the downward-slope was not significantly different between the groups (S_d_ = 198±16 fF/s in control cells; S_d_ = 161±11 fF/s in blebbistatin-treated cells) (Figure 1F).

The number of exocytic events observed in the perforated-patch recording and induced by stimulating with compound 48/80 was similar in control cells (63.1±4.5%) and cells under NM-2 inhibition (68.3±5.4%). However, the number of flicker events was reduced in blebbistatin-treated cells (12.1±1.8%) compared to control cells (25.6±4%) (Figure 1G). The number of endocytic events was not significantly different between the two groups. However, the downward steps increased by 83% when the inhibitor was present (11.3±2.5% versus 20.7±4.5%).

**Figure 2:**
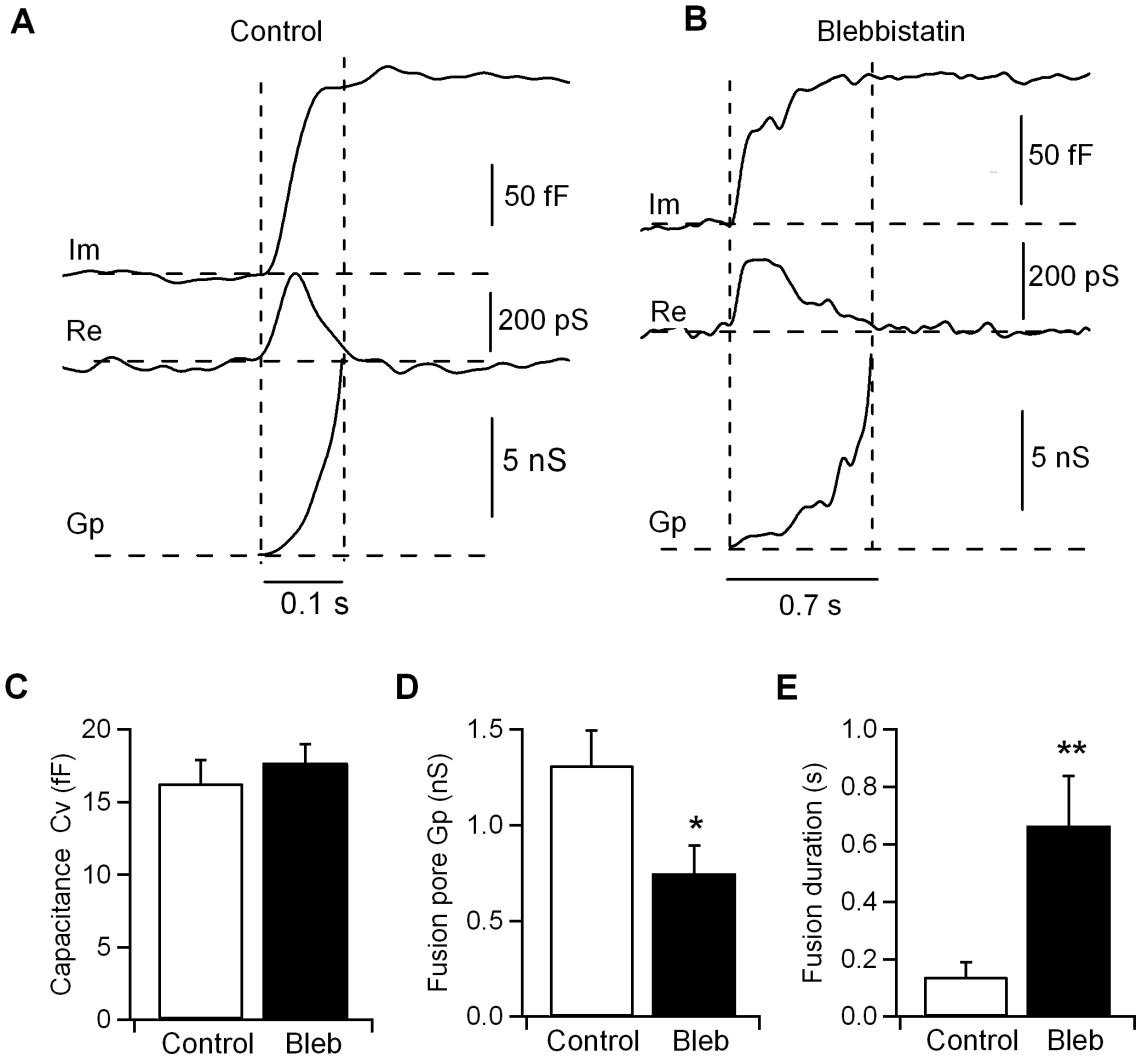
Effects of the NM-2 inhibitor blebbistatin on fusion pore kinetics in mast cells. Representative fusion pore events from control cells (A) and cells treated with 5 µM blebbistatin (B). The traces from top to bottom are the time course of the imaginary part of the admittance change (Im), the real part of the admittance change (Re) and the fusion pore conductance (Gp). The horizontal and vertical dashed lines indicate the baselines of the respective signals and the duration of the fusion pore, respectively. The mean capacitance step size of the endocytic vesicles was indistinguishable between control cells and cells treated with 5 µM blebbistatin (control: n = 547 events; blebbistatin: n = 582 events; p>0.05). C_v_, vesicle capacitance (C). A fusion pore analysis demonstrated that treatment with 5 µM blebbistatin decreased the fusion pore conductance Gp (D) and increased the fusion pore duration (E) (control: n = 35 pores; blebbistatin: n = 30 pores). Error bars, S.E.M. *p<0.05; **p<0.01.

For a small subset of individual exocytic events, the increase in the imaginary (Im) trace was associated with a detectable change in the real (Re) trace, reflecting the detection of a narrow fusion pore (Figure 2A), which was characterized by the fusion pore conductance (Gp) and duration. From the Im and Re traces, the time courses of Gp were calculated. We defined fusion pore expansion as full when Gp was > 2 nS. We then examined the fusion pore kinetics of exocytic events in cells treated with 5 µM blebbistatin. Our results showed that cells treated with this inhibitor displayed no changes in the capacitance step size of the exocytic events (16.3±1.6 fF; n = 547 in 23 control cells and 17.7±1.7 fF; n = 582 events in 19 cells inhibited with blebbistatin) (Figure 2C). As in control cells, the fusion pore conductance in blebbistatin-treated cells grew above 2 nS (Figure 2B), indicating that the fusion pore is opened [25], [26] and the vesicle fuses fully with the plasma membrane. However, the fusion pore Gp was reduced in blebbistatin-treated cells (0.75±0.15 nS; n = 30 pores) compared to control cells (1.3±0.2 nS; n = 35 pores) (Figure 2D), while the fusion pore duration was shorter in control cells (0.14±0.05 s) than in blebbistatin-treated cells (0.67±0.17 s) (Figure 2E), indicating a requirement for NM-2 in fusion pore dynamics. Consistent with these results, the overexpression of an nonphosphorylatable form of NM-2 in chromaffin cells has shown that this protein is an important factor in the rapid kinetics of pore expansion [12].

**Figure 3:**
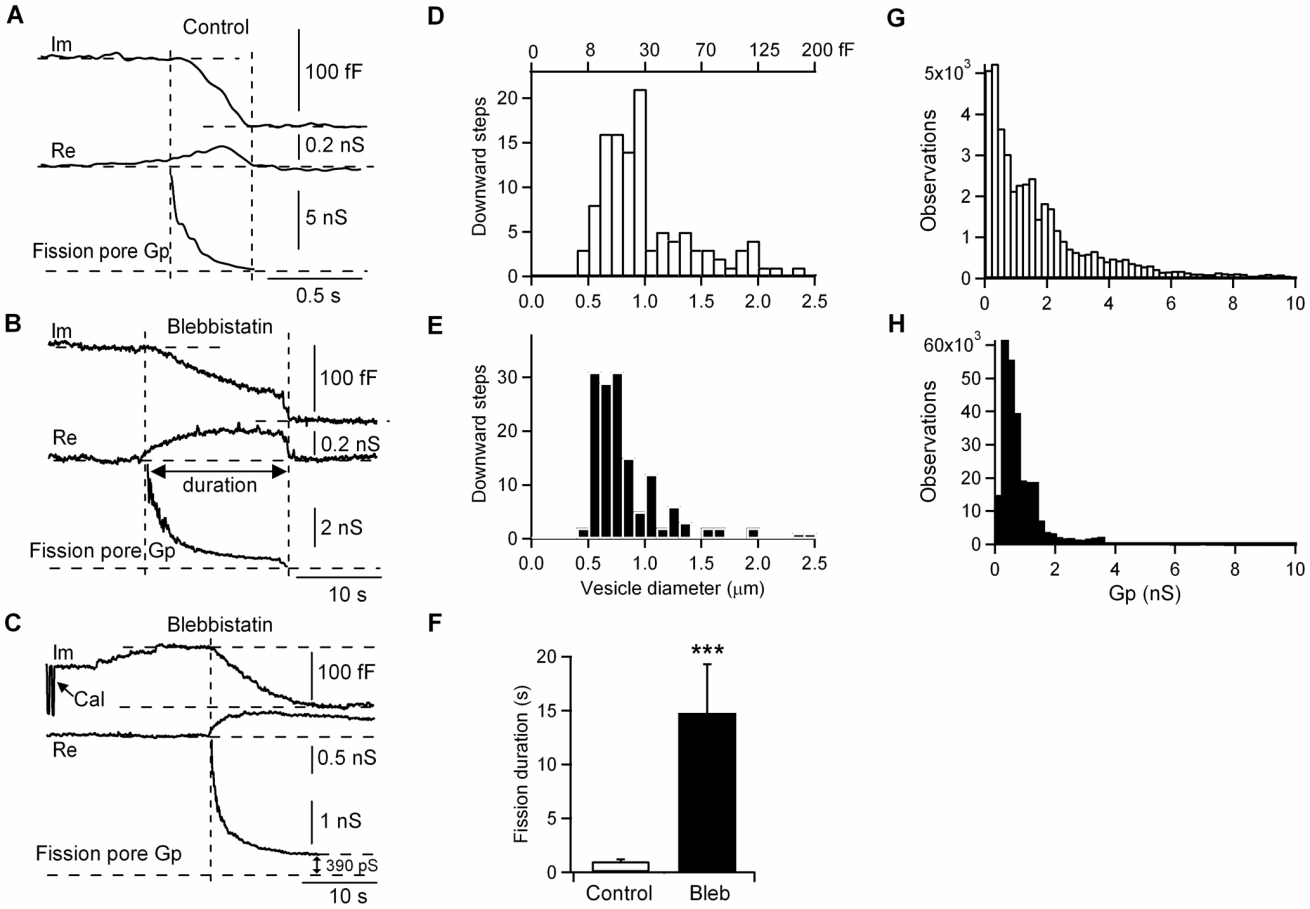
Effects of the NM-2 inhibitor blebbistatin on fission pore kinetics in mast cells. Representative fission pore events from control cells (A) and cells treated with 5 µM blebbistatin (B, C). The traces from top to bottom are the time course of the imaginary part of the admittance change (Im), the real part of the admittance change (Re) and the fission pore conductance (Gp). The horizontal and vertical dashed lines indicate the baselines of the respective signals and the duration of the fission pore, respectively. Two types of individual endocytic events were detected using perforated-patch capacitance measurements under NM-2 inhibition: a long event with detectable membrane scission (B) and an endocytic event without detectable pore closure, i.e., when a vesicle tries to undergo fission without closing completely (C). The change in the real trace during this type of event cannot be the result of an incorrect phase setting as demonstrated by the separation of Im and Re during a pure capacitance increase of 100 fF that was applied for online phase calibration (start of Im trace; Cal). The distribution of vesicle diameters were derived from endocytic capacitance step sizes from control cells (D, white) and blebbistatin-treated cells (E, black). Large downward steps associated with the retrieval of large areas of membrane (greater than one vesicle) were largely abolished (control: n = 124 events; blebbistatin: n = 148 events). Blebbistatin produced an increase in the fission pore duration compared to control cells (F). Frequency distribution of Gp values during fission pore closure of endocytic events from control cells (G, white) and blebbistatin-treated cells (H, black). Error bars, S.E.M. ***p<0.001.

Given the capability of this molecular motor to act on fusion pore expansion [12], [13], [27], we tested whether NM-2 has a direct role in vesicle fission during endocytosis by examining the fission pore kinetics of endocytic events in cells inhibited with blebbistatin (Figure 3). Typically, the fission pore closure manifests itself as a transient increase in the real part (Re) of the membrane admittance on a time scale of 100–3000 ms and is associated with a time-resolved decrease in the imaginary part (Im) (Figure 3A). Under blebbistatin inhibition, the durations of the transients in the Re trace were usually longer than 3000 ms (Figure 3B), and long transients that increased gradually without returning to the baseline were frequent (54% of pores) (Figure 3C). The example shown in Figure 3C illustrates the unsuccessful formation of an endocytic vesicle. A downward step is accompanied by a change in the Re trace; however, instead of being transient and vanishing when the vesicle membrane is excised from the plasma membrane and the fission pore is completely closed, the Re deflection reaches and remains at a stationary value when the capacitance step drops to the minimum level. The resulting pore conductance reveals the formation of a large vesicle that does not close completely and whose capacitance size remains unknown.

The mean step size of the downward steps was 35.5±2.9 fF (n = 124 events) in control cells and 27.7±2.6 fF (n = 148 events) in blebbistatin-treated cells, suggesting a significant change in vesicle size (p<0.05). The distributions of the endocytic capacitance step size and vesicle diameter were also different between control (Figure 3D) and blebbistatin conditions (Figure 3E), indicating a reduction in the number of large, but not small, endocytic events in cells lacking NM-2 function. Thus, the effect of blebbistatin on endocytosis is due to a decrease in the number of large events.

**Figure 4:**
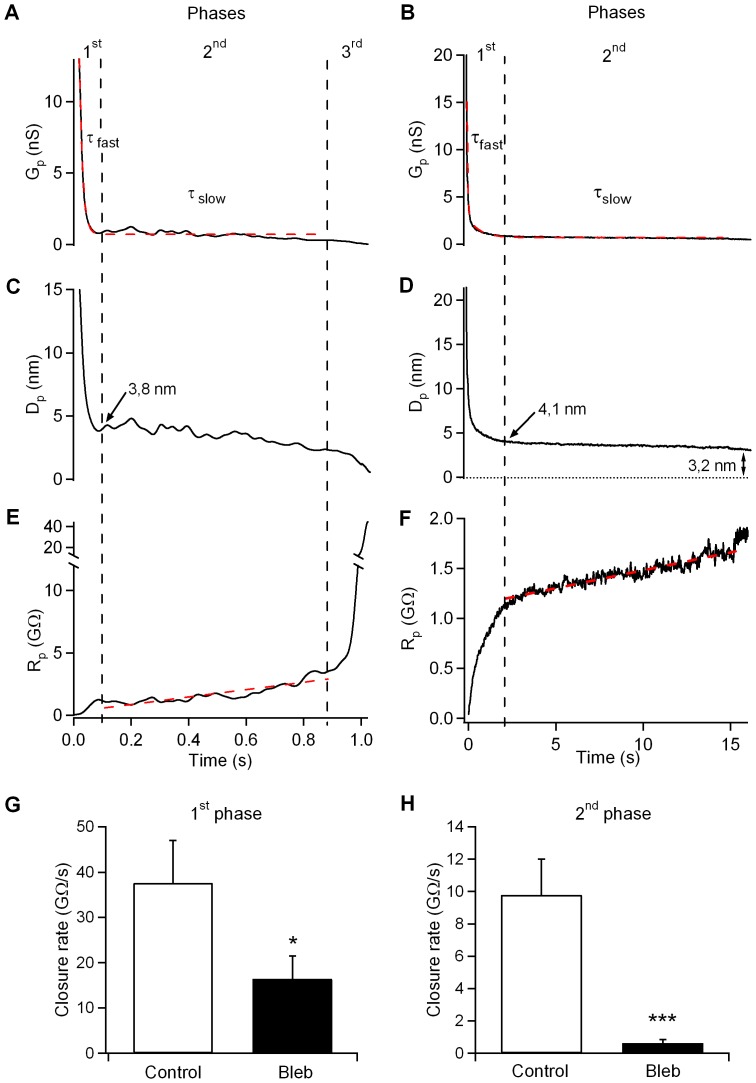
Blebbistatin affects fission pore dynamics. The time–course of Gp showed three different kinetic phases in control cells (A). A double exponential function depicts the two initial phases (red dashed line). A more pronounced Gp decrease accounts for the third phase (not appreciable in this magnification). This last phase is not present in the fission pore from the cell treated with blebbistatin (B). Fission pore diameters (Dp) from control (C) and treated cells (D) were calculated from the conductance data with the assumption that all conductance decreases result from changes in the diameter (assuming a pore length of 15 nm, which corresponds to the thickness of two membrane bilayers). A time–course of fission pore resistance (Rp) was calculated from the conductance data (E and F). The second phase could be fitted to a straight line in both the control pore (E) and the pore inhibited with blebbistatin (F) (dashed line; Pearson’s correlation coefficients: 0.89 and 0.95, respectively). The sudden increase in pore resistance indicated that the final pore closure (third phase) was absent from the pores in most cells lacking NM-2 function. The pore closure rates during the first (G) and second (H) phases in control conditions were higher than blebbistatin-treated pores. Error bars, S.E.M. *p<0.05; ***p<0.001.

The dynamics of fission pore closure reflect the molecular events leading to fission. Fission pore dynamics were quantified for large endocytic vesicles by measuring the lifetime of each fission pore and the fission pore conductance. Our results showed that the duration of fission pore closure was longer in cells lacking NM-2 function (control, 1.0±0.2 s, n = 46 events; Bleb, 14.7±4.5 ms, n = 26 events; p<0.001), indicating slow fission pore closure (Figure 3F). To define better the effects of blebbistatin on fission pore properties, we plotted the frequency distribution of Gp values measured during fission pore closure. The frequency distribution shows a large peak around 300–400 pS, indicating that the fission pore keeps longer in a partly opened state in blebbistatin-treated cells (Figures 3G and H). Figure 4 shows typical traces of the fission pore conductance (Gp), fission pore diameter (Dp) and fission pore resistance (Rp) of fission events in control and blebbistatin-treated cells. In control cells, fission pore closure was more rapid, and most fission events closed rapidly (*<*1 s). A typical fission pore is analyzed in Figure 4A. This event lasted ∼ 1 s, and analysis of the Gp revealed that it can be best fit to a double exponential, reflecting that the decrease in Gp occurs in phases. In the initial phase, the conductance decreases rapidly from 12 to 2 nS for approximately 40 ms, and then a slower decrease with an overall slope of approximately 1 pS/ms occurs. Finally, the Gp decreases more steeply. We used Gp to estimate the fission pore diameter (Dp) with the assumption that all conductance decreases are due to changes in diameter (Figure 4C). The initial Dp (∼ 15 nm) decreased to a final Dp of 3.8 nm during the first phase of Gp decay. Second, the Dp decreased very slowly and approached zero. The time–course of the fission pore resistance (Rp) (Figure 4E) reveals a linear trend increase in the second phase that suggests elongation of the pore at a constant speed. Finally, a sudden increase in the resistance indicates fission pore closure. The model that best explains the time-course of Gp has been previously defined by three stages: 1) a rapid reduction in diameter, 2) a relatively prolonged time period where the pore grows in length and 3) a final rapid closure phase [28]. However, in cells treated with blebbistatin, we observed a significant number of fission pores (14 of 26 pores) that exhibited longer lifetimes without the last phase of final closure, namely uncompleted fission pores. A fission pore from a blebbistatin-treated cell is shown in Figures 4B, D and F. This event lasted 15.9 s and shows a slow and steady increase in fission pore resistance, but the abrupt final increase that is observed in control pores was absent here. In addition, the pore diameter decreased but remained at a final size of 3.2 nm.

We further analyzed the effect of blebbistatin by examining which phase of the fission pore closure kinetics is affected. The rate of pore closure during the first phase was 37.6±9.4 GΩ/s (n = 46) in control cells. This rate was significantly reduced in cells inhibited by blebbistatin (16.4±5.1 GΩ/s; n = 26; p<0.05) (Figure 4G). During the second phase, the Rp in control cells can be fitted with a straight line and an average slope of 9.8±2.2 GΩ/s (Figure 4H). This rate is significantly faster than the rate observed with blebbistatin (0.6±0.2 GΩ/s; p*<*0.001). Together, these data suggest that blebbistatin has a predominant effect on the fission pore lifetime and determines the final membrane excision.

### NM-2 inhibition blocks vesicle membrane recapture following exocytosis

**Figure 5:**
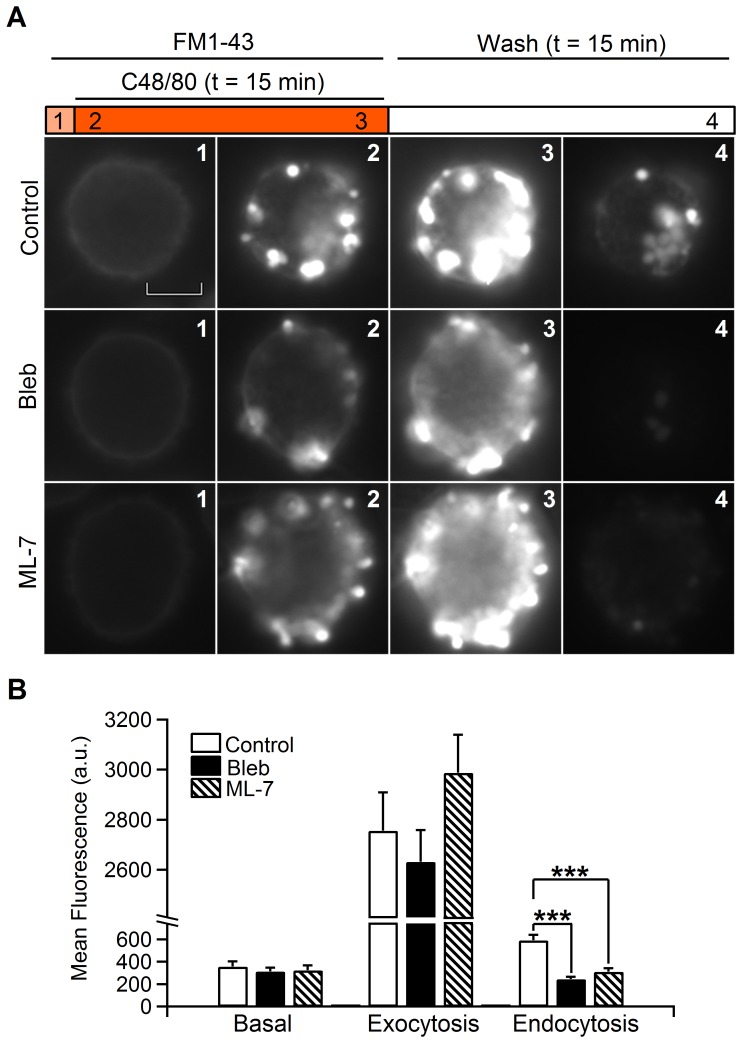
Inhibition of NM-2 activity through drug treatments produces decreased FM1-43 internalization. The standard protocol begins with an initial FM1-43 (4 µM) perfusion to label cells, followed by perfusion of C48/80 (100 µg/ml) in the presence of FM1-43 to stimulate secretion for 15 min. Finally, the extracellular FM1-43 label was removed by washing with a standard solution for 15 min (A). Cells that were treated with blebbistatin (5 µM) or ML-7 (5 µM) were previously incubated in a solution containing these drugs for 10 min. Panel A shows FM1-43 fluorescence images taken from control (Control) and Blebbistatin- (Bleb) or ML-7 (ML-7)-treated cells. From left to right: basal (1), at the beginning of the exocytosis (2), at the end of the exocytosis (3) and after removal of the extracellular dye (4). Internalized dye-labelled endocytic vesicles are evident as fluorescent spots within each cell (4). Mean fluorescence (arbitrary units, a.u.) data showing that basal and exocytic fluorescence (white bar, control; black bar, blebbistatin; and lined bar, ML-7) are equal for treated and non-treated cells. However, endocytic fluorescence was significantly lower for cells treated with blebbistatin or ML-7 (B). Scale bar: 5 µm. Error bars, S.E.M. ***p<0.001.

Previously, we established that mast cell vesicles stain with the fluorescent dye FM1-43 [4]. FM1-43 and similar styryl dyes have proven useful as probes for membrane trafficking because they reversibly stain membranes, are impermeable to membranes, and are more fluorescent when bound to membranes than when in solution. Upon stimulation, exocytosis causes vesicular membrane to fuse with cellular membrane, and dye binds to exocytosing membrane. Stained membrane is then endocytosed, and after washing out excess dye from the membrane, endocytosed vesicles can be observed [29]–[31]. During cell stimulation with compound 48/80 in the presence of FM1-43, vesicles fuse with the plasma membrane and release their contents; however, FM1-43 may be retained in the dense cores that are exposed to the extracellular space, allowing visualization of individual exocytic vesicles as bright spots, such as reported previously in lactotrophs [32]. Figure 5 shows control, blebbistatin-treated and ML-7-treated cells exposed to FM1-43 (Videos S1, S2 and S3). ML-7 is an inhibitor of the myosin light chain kinase [14]. In typical cells, stimulation caused a massive secretory response that was observed as intense and extensive fluorescence due to many single spots spreading progressively from the cell periphery toward the cell interior and sideways (Figure 5A). The fluorescent spots persisted for the duration of the experiments. When FM1-43 was washed from the bath, the dye was not destained completely, and some fluorescence signals remained associated to the same location of the previous spots, indicating the retrieval of FM1-43-stained dense cores. We measured the level of fluorescence in mast cells before and after destaining (Figure 5B). In control and treated cells, the mean fluorescence levels before (control, 358±47 a.u.; Bleb, 314±34 a.u.; ML-7, 324±44 a.u.) and after exocytosis (control, 2,757±153 a.u.; Bleb, 2,632±127 a.u.; ML-7, 2,988±152 a.u.) were approximately the same (p>0.05), indicating normal release. However because the fluorescence after destaining in the control cells (592±48 a.u.; n = 32 cells) was higher than in the treated cells (Bleb, 246±21 a.u.; n = 63 cells and ML-7, 310±30 a.u.; n = 43 cells; p<0.001), the amount of FM1-43 retrieval was reduced under NM-2 inhibition, suggesting that a smaller number of vesicles were retrieved.

**Figure 6:**
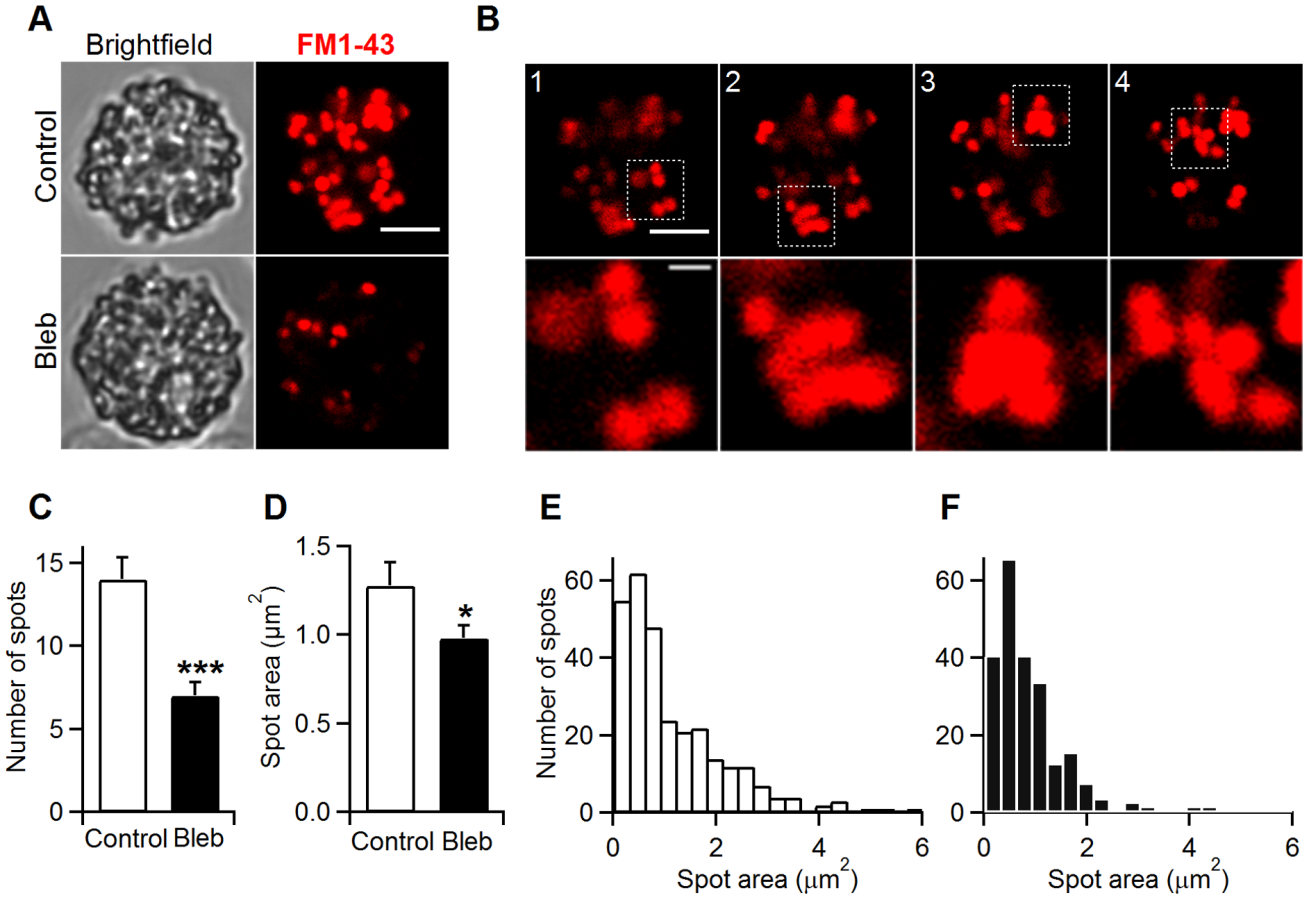
Compound endocytosis is inhibited by blebbistatin. The left images show brightfield single confocal sections, while the right images show Z-stack projections of endocytic spots labelled with FM1-43 (A). The data are from control cells (Control) and cells treated with blebbistatin (Bleb) (5 µM). In panel B, the upper pictures (1–4) show a sequence obtained gradually (at 2 µm separation) from the control cell shown in A. The lower pictures show an enlarged view of groups of endocytic vesicles (enclosed by a box in images 1–4) (B). A bar graph shows that the number of FM1-43 spots per cell in control (white) and blebbistatin conditions (black) was significantly different (C). The area of FM1-43 spots per cell was significantly reduced by blebbistatin (black) compared to control cells (white) (D). Histograms of the spot area for control conditions (E, white) and blebbistatin treatment (F, black). Scale bars: 5 and 1 (insets) µm. Error bars, S.E.M. *p<0.05; ***p<0.001.

To investigate the mechanism by which NM-2 contributes to endocytosis, we performed imaging experiments using confocal fluorescence microscopy with the styryl dye FM1-43. Following the same protocol as in Figure 5A, the cells were stimulated for 15 min and then washed to promote the uptake of FM1-43 dye into internalized vesicles. As expected, only a few fluorescent spots were observed under NM-2 inhibition (control, 14±1.3 spots/cell, n = 22 cells and Bleb, 7±0.8 spots/cell, n = 33 cells; p<0.001) (Figures 6A and C). This decrease in the number of endocytosis using FM1-43 in cells treated with blebbistatin don’t agree with capacitance data. Capacitance measurements allow to detect the formation of an endocytic vesicle as a downward step (Figure 1). Therefore, the number of capacitance downward steps usually reflects the number of endocytic events. However, the analysis of the fission pore conductance that reveals the dynamics of individual fission pore closures indicates that a 54% of resolved fission pores in the presence of blebbistatin were unclosed (Figure 3). According to this proportion, capacitance and fluorescence observations agree very closely (Table 1).

**Table 1:** Number of endocytic vesicles measured by capacitance and fluorescence measurements.

	Capacitance		Fluorescence
Treatment	Downward steps	Closed vesicles	FM-labelled spots
Control	17±3.8	17 (100%)	14±1.3
Bleb	14±1.9	6 (46%)	7±0.8

Comparing number of endocytic vesicles between capacitance (n = 23 cells, Control; n = 19 cells, Bleb) and fluorescence data (n = 22 cells, Control; n = 33 cells, Bleb) in controls and blebbistatin-treated cells. Downward steps include irreversible and reversible events. Numbers of closed vesicles were estimated from fission pore conductance data. Numbers are presented as means ± SEM per cell. Bleb, Blebbistatin.

To estimate the size of the internalized membrane, we next measured the area of the spots at different single sections obtained in the Z plane. In control cells, a large number of fluorescent spots showed an increase by two, three or greater fold of the area of a single spot (Figure 6B), indicating the compound endocytosis of several vesicles by a single fission event. The size of the fluorescent spot area varied but was smaller in treated cells than in control cells (control, 1.28±0.13 µm^2^, 257 spots, n = 22 cells; Bleb, 0.98±0.07 µm^2^, 237 spots, n = 29 cells; p<0.05) (Figure 6D). The distribution of the spot area showed that large endocytic spots greater than 2 µm^2^ were almost non-existent in blebbistatin-treated cells (Figure 6E and 6F). Interestingly, these data are consistent with the distribution of the downward steps as measured by capacitance (Figure 3D and E). Together, these results suggest that the inhibition of endocytosis by blebbistatin primarily affects the mode of compound endocytosis.

### Blebbistatin hinders complete vesicle closure

**Figure 7:**
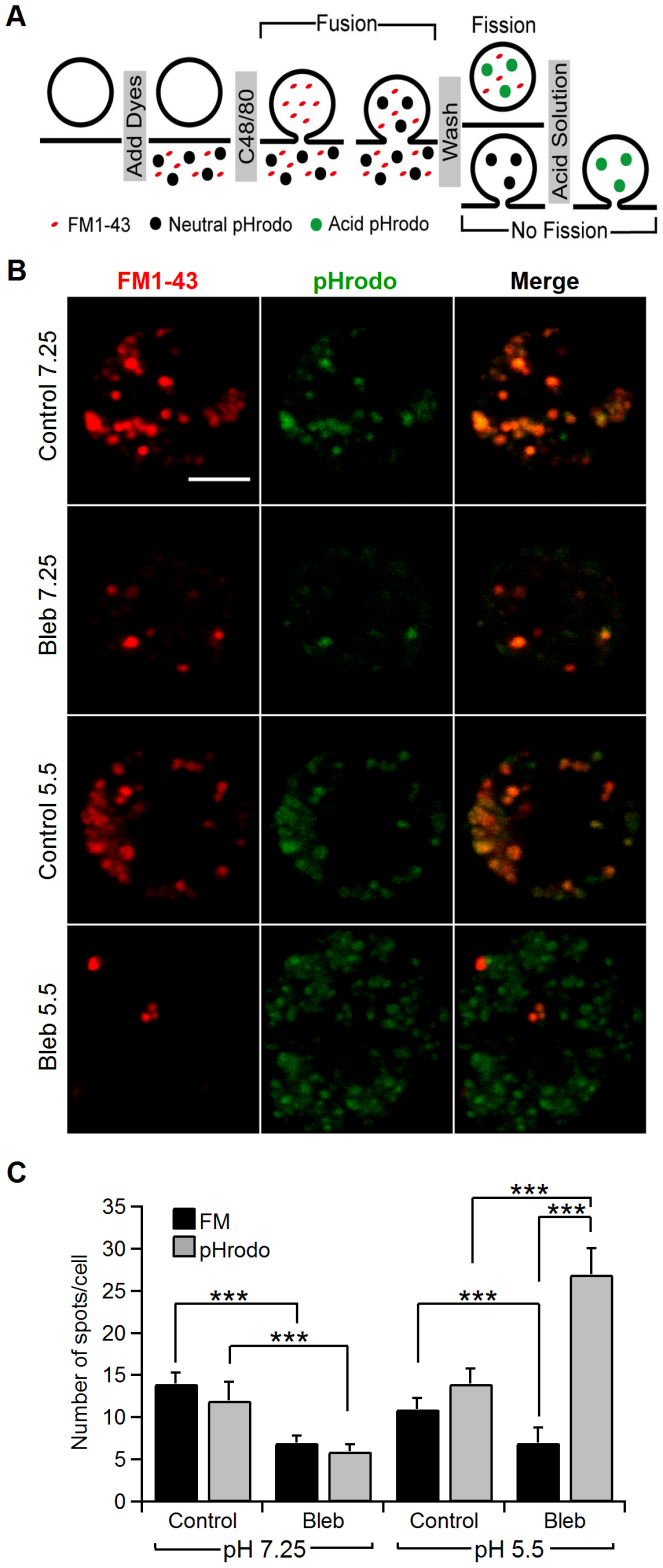
NM-2 inhibition blocks membrane scission and promotes unclosed vesicles through a narrow neck. Schematic of endocytic spots labelled with FM1-43 and pHrodo Green (A). After addition, the dyes incorporate into exocytic vesicles, which fuse with the membrane upon a stimulus (C48/80). If these vesicles are internalized and acidified, the vesicles will be stained. Therefore, after removing the extracellular dyes, only fluorescent endocytic vesicles remained. The addition of an extracellular acidic solution reveals the presence of uncompleted fission pores. The images in panel B show Z-stack projections from control cells (Control) or cells treated with blebbistatin (5 µM) (Bleb) at pH 7.25 or pH 5.5 after stimulation by C48/80 (100 µg/ml) (B). The stainings from left to right are: FM1-43 (red), pHrodo Green dextran (green) and a merge of FM1-43 and pHrodo Green dextran. The mean number of FM1-43 (black) and pHrodo Green dextran (grey) spots per cell in control conditions at pH 7.25 or pH 5.5 and after treatment with blebbistatin at pH 7.25 or pH 5.5 (C). Scale bar: 5 µm. Error bars, S.E.M. ***p<0.001.

We next investigated the incomplete fission events using FM1-43 and pHrodo Green dextran simultaneously. pHrodo Green is a 10,000 MW dextran-conjugated, pH-sensitive form of rhodamine that exhibits green fluorescence after endocytosis into the acidic interior of vesicles. We stimulated the cell preparation with compound 48/80 for 15 min in the presence of FM1-43 and pHrodo. After washing with standard solution (pH = 7.25), bright spots reflecting internalized vesicles were observed in red and green images and overlapped very closely (Figure 7B). As expected, the number of fluorescent spots with both FM1-43 and pHrodo at pH 7.25 in the presence of blebbistatin was scarce (FM1-43 control, 14±1.3 spots/cell; pHrodo control, 12±2.2 spots/cell, n = 22 cells; FM1-43 bleb, 7±0.8 spots/cell; pHrodo bleb, 6±0.8 spots/cell, n = 33 cells). However, when we used an acidic extracellular solution after removing the dyes, we were able to detect a large number of green spots in blebbistatin-treated cells, even greater than the control cell responses (FM1-43 control, 11±1.3 spots/cell; pHrodo control, 14±1.8 spots/cell; n = 27 cells; FM1-43 bleb, 7±1.8 spots/cell; pHrodo bleb, 27±3.1 spots/cell; n = 26 cells) (Figures 7B and C). A likely explanation is that the pHrodo dye may be retained inside unfissioned vesicles by a narrow fission pore in cells treated with blebbistatin. At neutral pH, these vesicles cannot be labelled, but at pH 5.5, the incomplete endocytosis is then revealed. The endocytic tubular neck formed during compensatory endocytosis under NM-2 inhibition acts as a molecular filter to retain the pHrodo dye while allowing the release of the FM1-43 dye. The diameters of the endocytic tubular necks should be between 1 and 5 nm. The smaller size would prevent the release of FM1-43 (diameter ∼ 1 nm), and the larger size would obstruct the release of pHrodo (diameter ∼ 5 nm). This interpretation agrees with the data obtained by the fission pore conductance analysis (Figures 3 and 4) and demonstrates that a high number of endocytic vesicles are unable to fully separate from the plasma membrane in cells treated with blebbistatin. In a similar way, when cells were treated with cytochalasin D, which inhibits actin assembly, the number of fluorescent spots was dramatically reduced, indicating a decrease in the incidence of scission (Table 2).

**Table 2:** Number of endocytic vesicles labelled with FM1-43 and pHrodo Green dyes.

	FM1-43-labelled spots/cell		pHrodo-labelled spots/cell	
treatment	pH 7.25	pH 5.5	pH 7.25	pH 5.5
Control	14±1.3	11±1.3	12±2.2	14±1.8
Bleb	7±0.8	7±1.8	6±0.8	27±3.1
variation	−50% (0.000)	−37% (0.000)	−50% (0.000)	+ 93% (0.001)
Cyto	4±0.9	4±0.9	1±0.3	14±2.9
variation	−71% (0.000)	−64% (0.000)	−92% (0.000)	0% (0.479)

Comparing control (22 cells at pH 7.25 and 27 cells at pH 5.5) to exposure to 5 µM Bleb (33 cells at pH 7.25 and 26 cells at pH 5.5) and to 4 µM Cyto (21 cells at pH 7.25 and pH 5.5). The pairs of data sets were compared using a two-tailed U-Mann-Whitney rank-sum test, and the result is indicated, between brackets, next to the variation of the mean. Numbers are presented as means ± SEM. Bleb, Blebbistatin; Cyto, Cytochalasin D.

## Discussion

**Figure 8:**
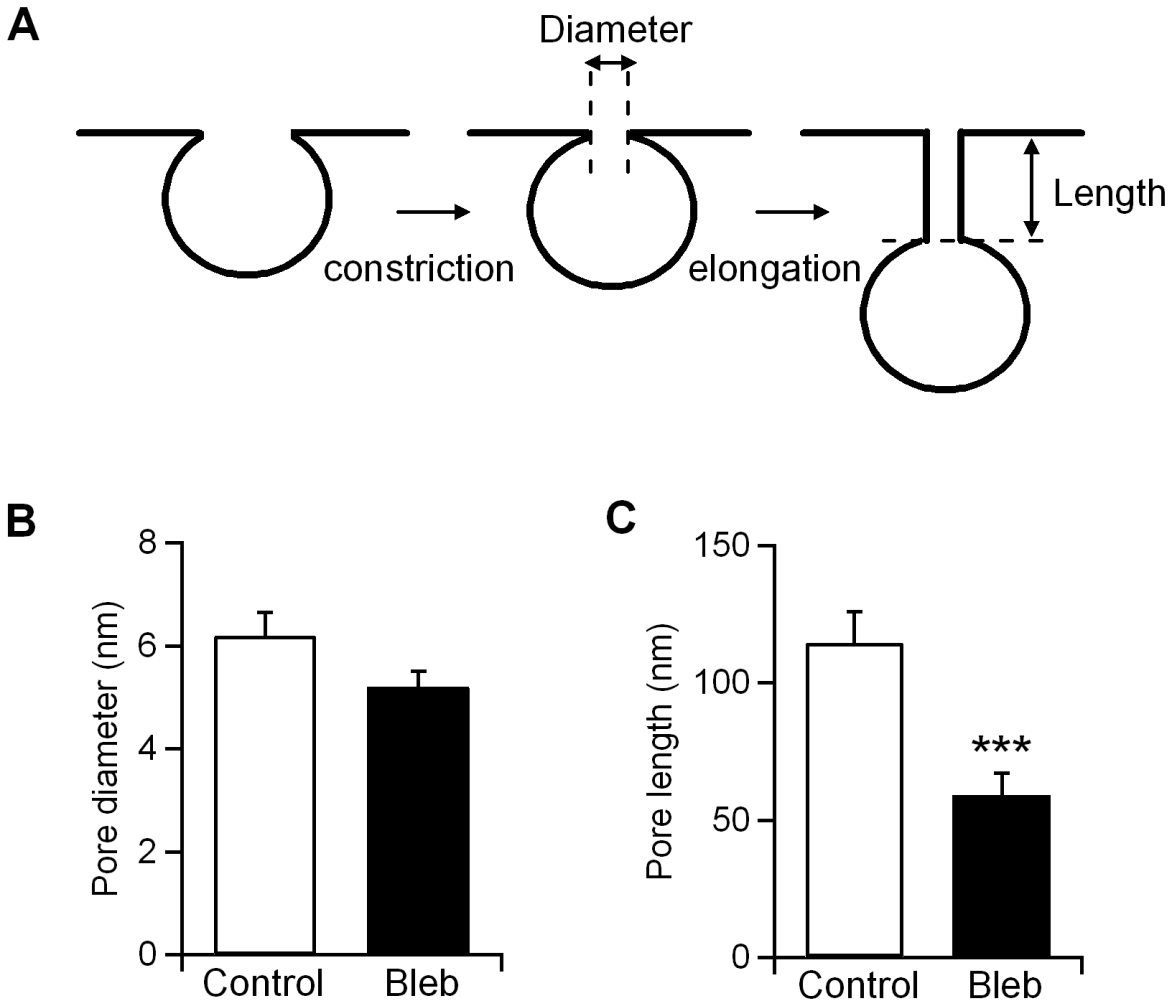
Blebbistatin affects fission pore geometry. A good model for explaining the dynamics of the fission pore is to assume that a decrease in pore diameter occurs initially (first phase) and is followed by a lengthening of the fission pore (second phase) (A) [28]. Assuming that successive steps of narrowing and lengthening explain the observed changes in pore resistance, we have calculated the final pore diameter (Dp final*)* and length (Lp final*)*. No significant differences were found in the final pore diameter between control and blebbistatin-treated cells (B). However, NM-2 inhibition reduced pore length (C). All error bars represent the S.E.M. ***p<0.001.

We have used capacitance measurements in the perforated-patch configuration together with fluorescence imaging of individual endocytic events labelled with the fluorescent dye FM1-43 and a pH-sensitive green fluorescent dextran (pHrodo) to measure the physical parameters associated with vesicle fusion and fission during exo-endocytosis in living cells. During exocytosis, our data indicated a decrease in the fusion pore conductance Gp and an increase in the pore duration by blebbistatin (Figure 2), confirming a role for NM-2 in regulating the secretory fusion pore [12], [13], [27]. Interestingly, we also present evidence that the NM-2 mediates vesicle scission during endocytosis in intact mast cells. An admittance analysis of our data revealed that fission occurs in three critical steps. These phases could be explained as pore constriction, pore elongation and pore collapse [28]. After formation, the endocytic vesicle neck (the fission pore) may rapidly constricts (first phase) to a specific, preferred shape and then elongate (second phase) (Figure 8A). In this model, no significant differences were identified in the final pore diameter obtained in control cells (6.2±0.4 nm) and blebbistatin-treated cells (5.2±0.3 nm) (Figure 8B), indicating that the final diameter of the pore was unaffected by NM-2. However, the geometric analysis showed that the fission pores tend to lengthen before final closure. Our observations indicate that blebbistatin reduces final pore lengthening (control, 114.7±11.2 nm; Bleb, 59±8.3 nm) and inhibits pore closing.

Some vesicles fused reversibly with the plasma membrane as indicated by an upward (exocytic) capacitance deflection followed by a downward (endocytic) event of similar magnitude (Figure 1). These kiss-and-run events accounted for 25.6% of the total events (black circles, Figure 1G). This value was reduced to 12.1% upon exposure of the cells to blebbistatin. Concomitantly, the number of downward steps was increased in the presence of blebbistatin (11.3% versus 20.7%). Surprisingly, the number of FM1-43 and pHrodo spots in cells exposed to the inhibitor at pH 7.2 was strongly reduced (Figure 6), suggesting that endocytosis was inhibited. This apparent discrepancy was resolved using acidic solution to discover the presence of pHrodo trapped inside frozen vesicles in a late step of endocytosis when the fission pore is formed and constricted (Figure 7). At pH 5.5, we were able to observe an even greater number of pHrodo spots in treated cells than in control cells in agreement with the electrophysiological data and likely reflecting a compensatory but unsuccessful cell mechanism to retrieve membrane. The exclusion of FM1-43, which is estimated to be ∼ 1.1 nm in size [33], from most endocytic vesicles and the accumulation of pHrodo Green dextran (10,000 MW), which is estimated to be ∼ 5 nm [34], in the endocytic vesicles of cells treated with the inhibitor at pH 5.5 suggest that the final neck diameter of vesicles that fail to close fully has an upper size limit of approximately 5 nm. This constricted pore is the fluorescent correlate of the ∼ 5.2 nm fission pore dimensions from endocytic events as estimated by conductance analysis (Figure 8). Because the final fission pore diameter seems not to be changed by the loss of NM-2 (Figure 8B), the present data support a role for NM-2 in the last stage of endocytosis to disconnect the vesicle completely. After narrowing, the late vesicle is pulled away from the cell surface, and the membranous neck that still connects the vesicle lengthens. Afterward, the neck is broken and the vesicle disconnects. Blebbistatin shortens the final pore length (Figure 8C). Therefore, based on our results, we propose a model that NM-2 is active at the site of vesicle fission through the dynamic re-arrangement of filamentous actin surrounding the vesicles. NM-2 is known to be required to coordinate actin assembly with coat compression and promote the retrieval of the exocytic granules [35]. It is possible that NM-2 exerts a tensional pressure in the F-actin network thereby affecting membrane tension, fission pore constriction or/and lengthening and finally fission of vesicle membrane. This action could exert a force that drives the vesicle away from the plasma membrane, increasing the strain on the constricted stalk until it severs. As a matter of fact, in culture mast cells, endocytic vesicles use actin polymerization to move into the citosol [36]. Alternatively, NM-2 might be able to influence the activity of the molecular machinery of endocytosis. In this sense, an association has been demonstrated between actin and dynamin [37]. On the other hand, when cells were treated with cytochalasin D endocytosis was drastically inhibited (71%). Nevertheless, when vesicles were subjected at acidic pH, the number of pHrodo-positive spots quite agreed with the number observed in control cells, revealing the involvement of F-actin in vesicle scission, according to the action observed by blebbistatin (Table 2). Actin polimerization plays a more general role in clathrin-mediated endocytosis. By using latrunculin, previous studies uncovered multiple aspects of clathrin-coated structure dynamics, including a marked reduction of approximately 80% in the incidence of scission [8], [9]. Curiously, staurosporine has also been observed to inhibit the separation of endocytic vesicles from the plasma membrane [38]. This dominant effect of staurosporine resembles the effect observed here by blebbistatin.

Furthermore, NM-2 may be especially relevant to mediate fission in compound endocytosis. This mechanism involves the recapture of the compound cavity that is formed by several fused vesicles in a sequential mode [4]. This mechanism was indicated by the analysis of capacitance measurements and FM1-43 fluorescence. The distribution of downward step sizes was shifted to the smaller values in cells treated with blebbistatin, suggesting a reduced number of large endocytic organelles (Figure 3). Additionally, a low number of compound FM1-43 spots were observed to inhibit NM-2 activity (Figure 6). Compound spots result from a chain or cluster of internalized vesicles through a single plasma membrane fission pore that disconnects the labelled lumen of these vesicles from the extracellular space. The present study indicates that compound endocytosis is a usual mechanism for membrane retrieval in mast cells and that NM-2 is essential for the direct retrieval of those large pieces of membrane generated by multivesicular fission. NM-2 inhibition does not completely block small vesicle endocytosis; this result indicates that additional factors intervene in vesicle fission. Likely, in the absence of NM-2, dynamin action may be sufficient to ensure the recapture of small vesicles by presently undefined mechanisms, which may include the recruitment of actin and proteins involved in actin polymerization [8], [9], [39], [40] and/or retrograde motor proteins, such as dynein [41]–[43]. Nevertheless, dynamin action may be ineffective to provide the force required to stretch the large membrane obtained from the cumulative fusion of several vesicles.

A recent report has shown that loss of NM-2 function results in significant decreases in the probability of clathrin-dependent internalization [44]. In the present study, we found that NM-2 inactivation inhibits vesicle closure, thereby increasing the number of incomplete endocytic events and reducing the number of kiss-and-run events (capacitance flickers) (Figure 1). This result correlates with the imaging data where 21±4% of the individual fluorescent spots containing FM1-43 result in a failure to accumulate pHrodo in the control cells. This percentage decreased to 11±3% in cells treated with blebbistatin (Figure S1). The size of the early fusion pore formed in reversible fusion was estimated to be ∼2 nm from the pore conductance analysis [4], [25], [45]. Accumulation of FM1-43 within a collapsed vesicle and the exclusion of pHrodo from the vesicle provide strong evidence for the formation of a reversible and narrow fusion pore (<5 nm). Because NM-2 activity may be required to mediate vesicle scission, the loss of activity may also contribute to preventing fusion pore closure in a reversible event, suggesting that at least the last phases of vesicle fission, i.e., membrane scission, occurs similarly in kiss-and-run endocytosis than in *de novo* endocytosis. This result contrasts with some reports where treatment with agents that inhibit myosin decreases the fusion pore duration and increases fusion pore closure [27], [46]. These contradictions most likely reflect discrepancies in the consequences of NM-2 action on both vesicle fusion and fission, as well as possible different dependencies of NM-2 on the vesicle size in many cell types.

The findings presented here show that in addition to its well established role in vesicle transport and exocytic fusion [15], NM-2 is also involved in vesicle fission. After formation, the fission pore may be constricted and lengthened before the final release of the vesicle into the cytoplasm. By treating with blebbistatin, the fission pore would be formed and constricted to a final size of approximately 5 nm, but lengthening and final scission would be blocked or dramatically slowed. These data showed that blebbistatin affected fission by causing incomplete vesicle closure and suggest a new role for NM-2 in endocytosis.

## Materials and Methods

### Animals and Ethics Statements

All experiments were performed according to guidelines from the European Community (Council Directive 86/609/EEC), the Spanish Real Decreto 223/1988, and Seville University regulations on laboratory animal care. The experiments were approved by the ethical committee of the University of Seville. Wild-type C57BL/6 mice were housed at regulated temperature (22±1°C) in a 12 h light/dark cycle with *ad libitum* access to food and drink. All efforts were made to minimize suffering. Mice were deeply anesthetized with CO_2_ exposure before decapitation.

### Cell preparation, reagents and materials

Peritoneal mast cells were prepared from 2- to 3-month-old mice following a procedure described in detail elsewhere [2], [47]. Briefly, cells were obtained by peritoneal lavage with a solution of the following composition (in mM): 140 NaCl, 45 NaHCO_3_, 10 HEPES, 3 KCl, 2 MgCl_2_, 1 CaCl_2_, 0.4 HNa_2_PO_4_ and 6 glucose. The cells were incubated at 37°C under 5% CO_2_ and 95% air atmosphere until use (between 1 and 6 h after culture). The external solution contained the following components (in mM): 140 NaCl, 10 HEPES, 3 KOH, 2 MgCl_2_ and 1 CaCl_2_. Glucose was added to adjust the osmolarity to 310 mOsmol.kg^−1^ (pH was adjusted to 7.25 or 5.5). Cytochalasin D inhibits actin polymerization. Stock solutions were prepared in dimethylsulfoxide at a final concentration of 4 mM (cytochalasin D), 1 mM (blebbistatin) and 10 mM 1-(5-Iodonaphthalene-1-sulfonyl)-1H-hexahydro-1,4-diazepine hydrochloride (ML-7) and were stored at −20°C. Compound 48/80 (C48/80) is a polymer that is known for its role in activating mast cells [48]. Stock solutions were prepared in water at a final concentration of 50 mg/ml and were stored at −20°C. All reagents and chemicals were obtained from Sigma-Aldrich with some exceptions: FM1-43 (Invitrogen-Molecular Probes, T35356) and pHrodo Green dextran (Invitrogen-Molecular Probes, P35368). The pH-sensitive rhodamine-based pHrodo Green dye has a pH-sensitive fluorescence emission that increases in intensity with increasing acidity. At extracellular pH 7.25, its fluorescence is diminished considerably. However, upon internalization, the acidic environment (pH≈5.5) of the vesicles elicits a bright green fluorescent signal from this dextran conjugate. This probe has been used to study autophagy [49] as well as to describe the sites and kinetics of vesicle release [50]. FM1-43 (1 mM) and pHrodo Green (0.5 mg/ml) stock solutions were aliquoted and store at −20°C, protected from light.

### Confocal microscopy

Isolated mast cells were exposed to C48/80 (100 µg/ml) along with FM1-43 (4 µM) and pHrodo Green (50 µg/ml) for 15 min. Then, the cells were washed with an external solution (pH 7.25 or pH 5.5). For treated preparations, the cells were previously incubated with blebbistatin (5 µM) or cytochalasin D (4 µM) for 10 min at room temperature. Live cell confocal microscopy was used to generate the imaging data. Images were captured using an upright Olympus FV1000 confocal laser scanning microscope equipped with three excitation laser lines (argon-krypton laser with 488, 561 and 633 nm excitation lines). Images were obtained using a 60X water immersion objective (82 nm/image pixel, NA 1.2) with similar conditions (laser intensities and photomultiplier voltages). During image acquisition (t = 8 µs/pixel), an alternating sequence of laser pulses at 561 and 488 nm wavelengths were used to illuminate FM1-43- and pHrodo-loaded vesicles, respectively. Fluorescence was filtered at 580−620 nm (FM1-43) and 500−560 nm (pHrodo Green), according to their emission wavelengths. All of our data are three-dimensional images reconstructed from 1 µm Z-stacks using ImageJ software (W. Rasband, National Institutes of Health, Bethesda, MD). To determine the endocytic spots, single slices from a Z-stack of each cell were analyzed. In this manner, duplicated spots found in more than a single slice were discarded. Endocytic spots were assumed to be simple (a single vesicle) or compound (a group of vesicles). A reversible fusion was defined when the spot only exhibited the FM1-43 label. The reversible fusion was quantified by determining the number of FM1-43-positive and pHrodo-negative spots with respect to the total number of FM1-43-labeled spots (all endocytic events). For detecting and measuring endocytic spots, we used HCImage software (Hamamatsu Photonics) to perform quantitative analysis on the raw images. The spots were selected based on threshold brightness and a minimum spot area. All experiments were performed at room temperature (22–24°C).

### Epifluorescence microscopy

Epifluorescence imaging was performed as described previously [4]. Briefly, experiments were conducted using an Axiovert 200 inverted microscope equipped with a Hamamatsu ORCA-R2 camera. All images were acquired using a 63X oil immersion objective (102 nm/image pixel, NA 1.3) and a standard filter set (XF115-2; Omega Optical). Exposures lasted for 0.2 s, and images were acquired at ∼ 1 Hz. External solutions were exchanged by a fast superfusion device that consisted of a multibarreled pipette. The common outlet of the pipette was positioned 50–100 µm from the cell. The standard and FM1-43 solutions were changed using a pinch valve controller system (Warner Instruments) that was manipulated by a manual switch. The flow rate (0.5–1 ml.min^−1^) was regulated by gravity to achieve a complete replacement of the cell surroundings in less than 2 s. All experiments were performed at room temperature (22–24°C). Cells that were treated with blebbistatin (5 µM) or ML-7 (5 µM) were incubated at room temperature in the presence of these reagents for 10 min immediately before recording, according to the standard protocol (Figure 5A).

Unprocessed images were analysed using HCImage software (Hamamatsu Photonics). Each cell was manually selected, and the average fluorescence was measured (basal, exocytosis and endocytosis fluorescence). All values were corrected by subtracting the background.

### Electrophysiological recordings

Electrophysiological recordings in this study were performed in the perforated-patch configuration. The perforated-patch pipette solution contained (in mM): 135 Cs-glutamate, 10 HEPES, 9.5 NaCl and 0.5 tetraethylammonium chloride. The solution was adjusted to pH 7.2 with CsOH. Pipettes with 2–5 MΩ resistance were pulled from borosilicate glass capillary tubes, fire-polished and partially coated with wax. Patched cells were allowed to perforate to less than 30 MΩ series resistance prior to recording. Perforated-patch capacitance was measured with a lock-in amplifier (SR-830; Stanford Research Instruments). Cells that were treated with blebbistatin (5 µM) were incubated for 10 min immediately before the recording. The C-slow and G-series potentiometers of an EPC-7 patch clamp amplifier (Heka Electronics) were used to cancel out the incoming membrane current to resolve small capacitance changes. We obtained a calibration signal by unbalancing the C-slow potentiometer by 100 fF, which corresponds to a change of 10 µm^2^ (assuming a specific membrane capacitance of 1 µF.cm^−2^). We set the phase manually at the beginning of each recording. The V-command was a 50 mV sine wave (root mean square, 1 kHz). The data acquisition was performed with a 16-bit A/D converter (6052-E; National Instruments) with locally written software in Igor Pro (Wavemetrics, Inc.). One data point was obtained every millisecond.

### Data analysis

**Figure 9:**
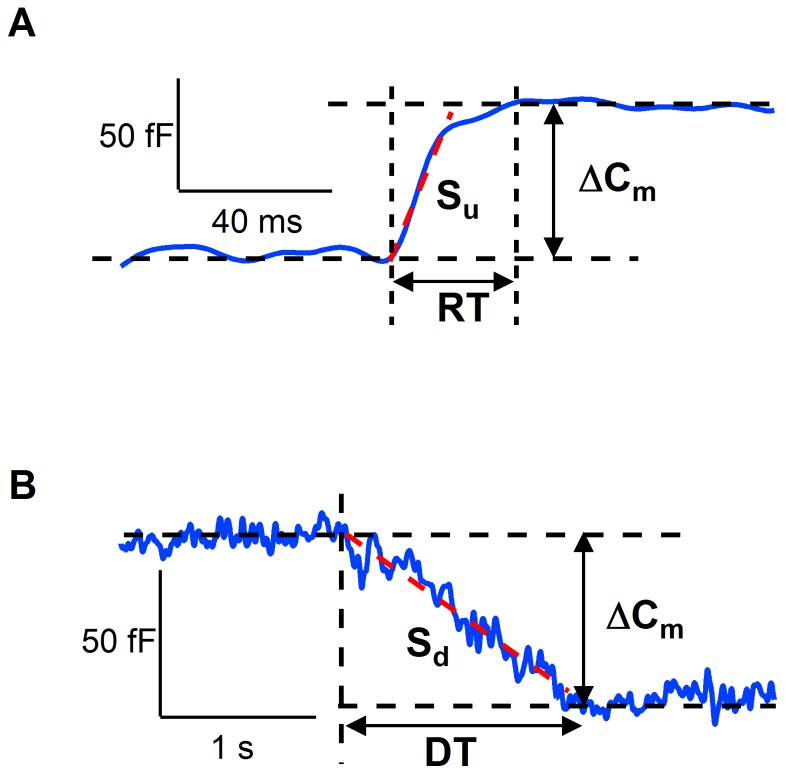
Capacitance parameters. The vertical distance at the step position defined the amplitude of ΔC_m_. The rise time (RT) or decay time (DT) shows the time required to reach the maximum or minimum amplitude, respectively, of the capacitance step, while the rate of membrane fusion and fission are measured as the slope of the linear fit to each upward (A) and downward (B) correspond to S_u_ and S_d_ respectively (red dashed lines).

The X and Y outputs of the lock-in amplifier reflect the real (Re) and imaginary (Im) conductance components. Online phase calibration resulted in good separation of the Im and Re conductance changes. The remaining small phase offsets were corrected offline [51]. When the Re is constant, the capacitance changes are reflected by Im/2πf, where f is the sine wave frequency. However, when Re changes transiently upon a capacitance step, which indicates a fission pore, the vesicle capacitance (Cv) and pore conductance (Gp) were calculated as described [52]. Data analysis was performed using macros for Igor (Wavemetrics Inc.). We analyzed the step and rise/decay time from each single exocytic/endocytic event (Figure 9). When a step was detected, regression lines were fitted through 100 ms segments both before and after the putative step. The noise about the regression lines and the step amplitude was measured. Steps were accepted if the amplitude exceeded the noise about the regression lines at least 3-fold. The duration of the event was taken as the time between the onset and the end of the capacitance step. The onset of the step was determined by the decay of the signal from the baseline by 3xRMS (root-mean-square) noise. The step endpoint was taken as the first point with a value that intercepts the baseline. The capacitance step size reflects the area of the fused and retrieved vesicles. The rise time (RT) and decay time (DT) indicate the fusion and fission pore lifetime, respectively, while the rate of membrane fusion and fission are measured as the slope of the linear fit to each upward and downward capacitance step, respectively. Vesicle radii were calculated by assuming that a vesicle is spherical and its specific membrane capacitance is 1 µF/cm^2^. Pore conductance (Gp) was calculated from the Re and Im parts of the admittance after baseline subtraction using the following equation (1): Gp =  (Re^2^ + Im^2^)/Re. Most of the fusion and fission pores were reliably detected for steps sizes > 50 fF, because the unavoidable noise of the RC circuit formed by access resistance and membrane capacitance [53], [54] requires large granules to obtain detectable signals. Individual steps smaller than 50 fF were not accompanied by a clear conductance change; however, exocytic/endocytic events larger than 50 fF were frequently accompanied by a transient change in conductance. The time constants (τ) of the Gp decreases were determined by fitting to a double exponential function. Gp was used to estimate the fission pore diameter [26], [55] using the equation (2): Dp =  (4ρL/π)^0.5^Gp^0.5^. The simplest model is a cylindrical pore where ρ, the bath solution resistivity, is a constant (100 Ω·cm) and L, the length of the pore, is commonly assumed to be a constant that is equivalent to the thickness of two membrane bilayers (15 nm). Resistance is directly proportional to the length of the pore and is also inversely to the area of a cross-section of the pore. Therefore, if the change in pore conductance were caused solely by an increased in pore length, the elongation would be proportional to an increase in pore resistance. The rate of fission pore closure was approximated from the slope of the linear fit to the resistance trace. Assuming that successive steps of narrowing and lengthening explain the observed changes in pore resistance, we have calculated the final pore diameter (Dp final) and length (Lp final). For each fission event, we converted the Gp trace into Dp and measured the pore size at the end of the first phase before the vesicle starts to elongate using equation (2). To find the pore length before scission, we measured the value of Rp before the final steep rise and calculated the pore length using equation (2).

### Statistics

All data are expressed as the mean ± S.E.M. Statistical significance was performed using a two-tailed, nonparametric Mann-Whitney test (samples were considered significantly different when p≤0.05) with SPSS Statistics 22.0 software.

## Supporting Information

Figure S1 **Blebbistatin reduces the number of reversible fusion events.** A schema of endocytic events labelled with FM1-43 and pHrodo Green (A). After addition, the dyes incorporate into exocytic vesicles, which fuse with the membrane upon a stimulus (C48/80). If the vesicle performs a reversible fusion, a narrow pore would be created (<5 nm) whereby only the FM1-43 can diffuse (lower cartoon). However, an irreversible fusion followed by a fission event would simultaneously exhibit FM1-43 and pHrodo staining (upper cartoon). Panel B shows a Z-stack projection obtained from a control cell. The left image corresponds to an overlay of FM1-43 and pHrodo Green fluorescence (merge). The upper panels show a fission event (1). This spot is simultaneously labelled with FM1-43 (red) and pHrodo Green (green). As a result, the overlap between the two images results in yellow fluorescence (merge). The lower panels show a reversible fusion event (2) where the fluorescent spot results from FM1-43 staining (left) but not pHrodo labelling (middle). Therefore, the spot does not exhibit yellow fluorescence (B). The number of spots labelled with FM1-43 but not pHrodo Green (reversible fusion) were quantified and are shown in the bar graph (control: 21±4%, n = 19 cells; Bleb: 11±3%, n = 27 cells) (C). Scale bars: 5 and 1 (insets) µm. Error bars, S.E.M. *p<0.05.
(TIF)

Video S1 **Exocytosis and endocytosis dynamics of a mast cell labelled with FM1-43.** Scale bar: 5 µm.
(ZIP)

Video S2 **Exocytosis and endocytosis dynamics of a mast cell treated with blebbistatin in the presence of FM1-43.** Scale bar: 5 µm.
(ZIP)

Video S3 **Exocytosis and endocytosis dynamics of a mast cell treated with ML-7 in the presence of FM1-43.** Scale bar: 5 µm.
(ZIP)
