# Supplementary figures and images for: A New Role for Myosin II in Vesicle Fission
Source: PLoS One. 2014 Jun 24;9(6):e100757. doi: 10.1371/journal.pone.0100757 (PMC4069105; doi:10.1371/journal.pone.0100757)

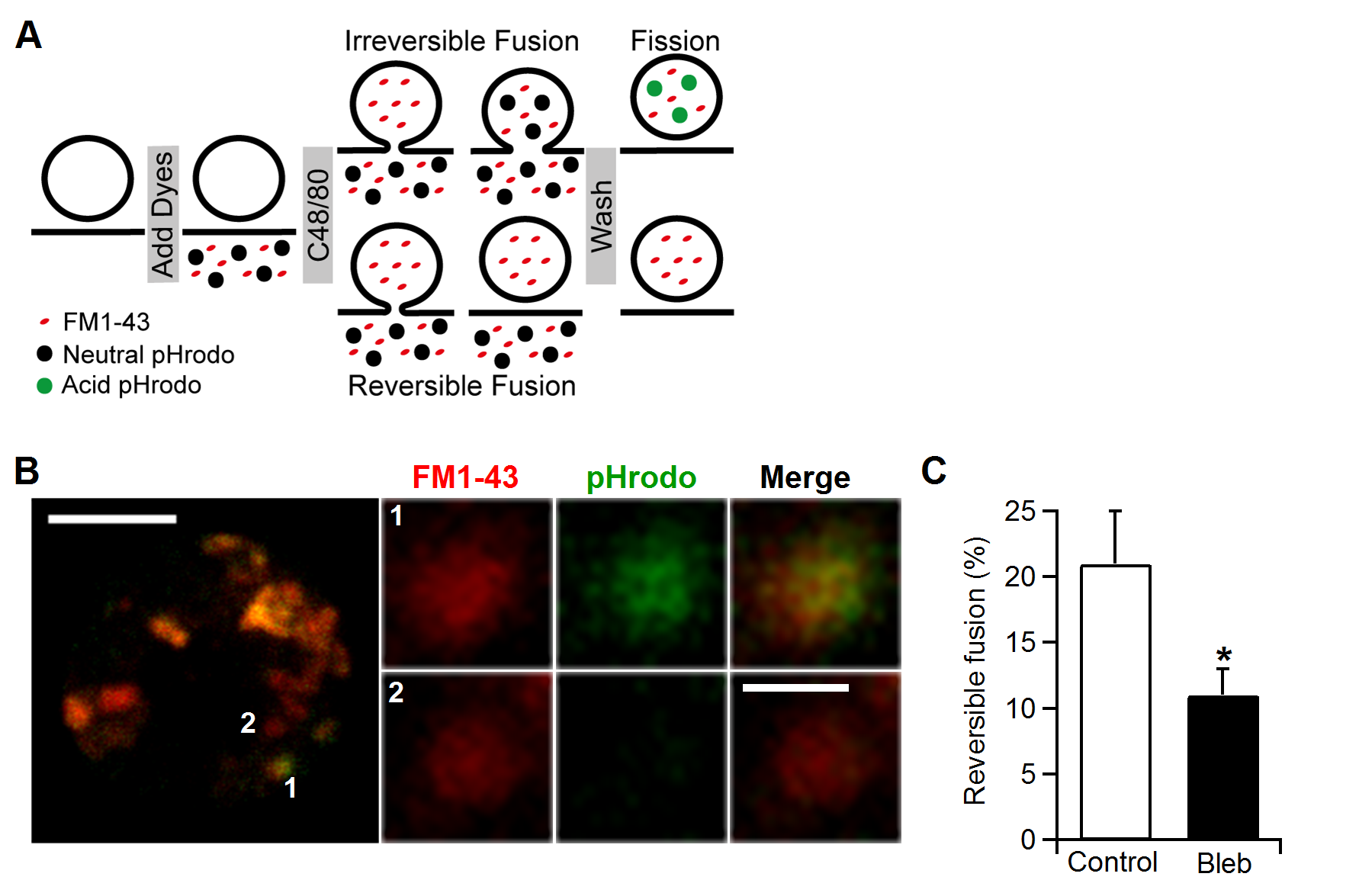

Supplement: Figure S1 — Blebbistatin reduces the number of reversible fusion events. A schema of endocytic events labelled with FM1-43 and pHrodo Green (A). After addition, the dyes incorporate into exocytic vesicles, which fuse with the membrane upon a stimulus (C48/80). If the vesicle performs a reversible fusion, a narrow pore would be created (<5 nm) whereby only the FM1-43 can diffuse (lower cartoon). However, an irreversible fusion followed by a fission event would simultaneously exhibit FM1-43 and pHrodo staining (upper cartoon). Panel B shows a Z-stack projection obtained from a control cell. The left image corresponds to an overlay of FM1-43 and pHrodo Green fluorescence (merge). The upper panels show a fission event (1). This spot is simultaneously labelled with FM1-43 (red) and pHrodo Green (green). As a result, the overlap between the two images results in yellow fluorescence (merge). The lower panels show a reversible fusion event (2) where the fluorescent spot results from FM1-43 staining (left) but not pHrodo labelling (middle). Therefore, the spot does not exhibit yellow fluorescence (B). The number of spots labelled with FM1-43 but not pHrodo Green (reversible fusion) were quantified and are shown in the bar graph (control: 21±4%, n = 19 cells; Bleb: 11±3%, n = 27 cells) (C). Scale bars: 5 and 1 (insets) µm. Error bars, S.E.M. *p<0.05. (TIF) [file pone.0100757.s001.tif]
